# Performance evaluation of ChatGPT, GPT-4, and Bard on the official board examination of the Japan Radiology Society

**DOI:** 10.1007/s11604-023-01491-2

**Published:** 2023-10-04

**Authors:** Yoshitaka Toyama, Ayaka Harigai, Mirei Abe, Mitsutoshi Nagano, Masahiro Kawabata, Yasuhiro Seki, Kei Takase

**Affiliations:** 1 Department of Diagnostic Radiology, Tohoku University Hospital, 1-1 Seiryo-Machi, Aoba-Ku, Sendai, 980-8575 Japan; 2 Department of Radiology, Tohoku Medical and Pharmaceutical University, Sendai, Japan; 3 Department of Diagnostic Radiology, Tohoku University Graduate School of Medicine, Sendai, Japan; 4 School of Medicine, Tohoku University, Sendai, Japan; 5 Department of Radiation Oncology, Tohoku University Hospital, Sendai, Japan

**Keywords:** ChatGPT, GPT-4, Bard, Japan Radiology Society

## Abstract

**Purpose:**

Herein, we assessed the accuracy of large language models (LLMs) in generating responses to questions in clinical radiology practice. We compared the performance of ChatGPT, GPT-4, and Google Bard using questions from the Japan Radiology Board Examination (JRBE).

**Materials and methods:**

In total, 103 questions from the JRBE 2022 were used with permission from the Japan Radiological Society. These questions were categorized by pattern, required level of thinking, and topic. McNemar’s test was used to compare the proportion of correct responses between the LLMs. Fisher’s exact test was used to assess the performance of GPT-4 for each topic category.

**Results:**

ChatGPT, GPT-4, and Google Bard correctly answered 40.8% (42 of 103), 65.0% (67 of 103), and 38.8% (40 of 103) of the questions, respectively. GPT-4 significantly outperformed ChatGPT by 24.2% (*p* < 0.001) and Google Bard by 26.2% (*p* < 0.001). In the categorical analysis by level of thinking, GPT-4 correctly answered 79.7% of the lower-order questions, which was significantly higher than ChatGPT or Google Bard (*p* < 0.001). The categorical analysis by question pattern revealed GPT-4’s superiority over ChatGPT (67.4% vs. 46.5%, *p* = 0.004) and Google Bard (39.5%, *p* < 0.001) in the single-answer questions. The categorical analysis by topic revealed that GPT-4 outperformed ChatGPT (40%, *p* = 0.013) and Google Bard (26.7%, *p* = 0.004). No significant differences were observed between the LLMs in the categories not mentioned above. The performance of GPT-4 was significantly better in nuclear medicine (93.3%) than in diagnostic radiology (55.8%; *p* < 0.001). GPT-4 also performed better on lower-order questions than on higher-order questions (79.7% vs. 45.5%, *p* < 0.001).

**Conclusion:**

ChatGPTplus based on GPT-4 scored 65% when answering Japanese questions from the JRBE, outperforming ChatGPT and Google Bard. This highlights the potential of using LLMs to address advanced clinical questions in the field of radiology in Japan.

## Introduction

Artificial intelligence (AI) has witnessed unprecedented advancements, such as the emergence of large language models (LLMs) [11]. Typically, LLMs employ a deep learning architecture to process natural language data on a large scale, and the potential applications of LLMs in medicine lie within the realm of research and education, as well as clinical practice, particularly as decision aids [23]. In clinical radiology practice, LLMs may be used as tools to obtain, supplement, and confirm relevant expert-level knowledge that could affect the clinical decision-making processes of radiologists. With the increased accessibility of LLMs facilitated by application programming interfaces (APIs), this trend must grow in the future when radiologists who perform keyword searches on books and websites start asking questions directly to LLMs.

Two LLMs that have demonstrated exceptional performance in the US Medical Licensing Examination (USMLE)-style questions, surpassing the approximate passing threshold of 60%, are GPT-4 and Med-PaLM 2 [13, 16, 17, 21]. GPT-4, a successor to ChatGPT using GPT-3.5, was developed by OpenAI and is currently accessible on ChatGPTplus [18, 19]. It offers prompt-based instant and informative responses and is capable of generating creative texts, such as novels and poems. The training data for both ChatGPT and GPT-4 exceeded 45 TB of text data until September 2021, although neither was fine-tuned for medical data [11, 19, 23]. By contrast, PaLM 2, whose fine-tuned medical version is Med-PaLM 2, is used in Google Bard and is capable of utilizing up-to-date data, extending its potential to maintain pace with the rapidly changing landscape of medicine [7]. Because Med-PaLM 2 is yet to be publicly available, Google Bard based on PaLM 2 was used in this study.

Although the outstanding performance of GPT-4 in radiology has already been established through benchmarking on the board-style examination of diagnostic radiology in Canada and the United States [8, 9], the capabilities of LLMs in predicting responses to specific questions in Japanese radiology clinical practice remain unclear. To address this gap, we focused on the Japan Radiology Board Examination (JRBE), a meticulously designed test renowned for its comprehensive coverage of clinical knowledge and applications, encompassing not only diagnostic imaging but also nuclear medicine and radiation oncology. This examination also included questions pertaining to the domestic healthcare landscape, including approved medications and medical procedures. Given that LLMs leverage transformer-based architectures to generate contextual responses by predicting upcoming words and phrases based on the preceding text [23], the use of Japanese prompts was chosen, with the expectation that this might facilitate more effective response alignment with Japan’s distinct healthcare context. The possible disadvantage of using Japanese prompts may be attributed to the typological distance between Japanese and English [15, 22], as well as the significantly lesser volume of available Japanese training data compared to the English counterpart [1]. In this study, we compared the overall and category-specific performance of ChatGPT, GPT-4, and Google Bard on the JRBE.

## Materials and methods

### Questions dataset

The questions used in this study were multiple-choice questions from the 2022 JRBE, which are accessible to Japan Radiological Society (JRS) members [2]. The JRS granted permission to utilize these questions. The JRBE is a cross-disciplinary radiology knowledge exam created for senior residents who have completed a three-year radiology residency program in Japan. In 2022, 232 out of 247 candidates passed the exam (93.9% of pass rate); however, the exact passing score was not disclosed. Of the 105 questions, one table question and one image question were excluded from the study as ChatGPT, GPT-4, and Google Bard did not recognize these styles. In total, 103 questions were included.

Because the official JRBE answers were unavailable, two board-certified radiologists (Y.T., a diagnostic radiologist with 12 years of experience; M.K., a diagnostic radiologist with 7 years of experience) and a radiology senior resident (Y.S., with 2 years of experience) independently reviewed and answered the questions, citing books and websites as needed. The answers were deemed correct when there was agreement. For disagreements, a single correct answer was selected by consensus.

### Question classification

In the study, the questions were classified by question patterns (i.e., the number of correct answers out of five choices), required level of thinking (lower or higher order), and topics. Of the 103 questions, 17 had two correct answers and three distractors (i.e., two-answer questions), whereas the remaining 86 had one correct answer and four distractors (i.e., single-answer questions). The principles of Bloom’s Taxonomy for Learning and Assessment [6, 10] were used to classify the questions by the required level of thinking. Among the 103 questions, 59 required lower-order thinking, necessitating recall and basic understanding. The remaining 44 questions required higher-order thinking skills, such as application, analysis, or evaluation. The questions were classified into the following five topics: diagnostic radiology (52), interventional radiology (3), nuclear medicine (15), radiation oncology (23), and general radiological knowledge (10).

### Data collection and assessment

The responses from ChatGPT, GPT-4, and Google Bard were collected between May and July 2023. The questions were manually entered into the text input area, which appears on the publicly accessible website of these models, one at a time in the order of examination using a single chat [3, 12]. No specific prompts were used. Each response was assessed by a radiologist (Y.T.), and only responses that stated the correct answers were scored as correct. For the two-answer questions, only cases in which ChatGPT, GPT-4, or Google Bard correctly chose both correct answers were scored as correct.

### Statistical analyses

The performance of ChatGPTplus based on ChatGPT, GPT-4, and Google Bard was analyzed using basic standard descriptive statistics, such as numbers, proportions, and 95% confidence intervals. McNemar’s test was used to compare the proportion of correct responses between two LLMs. To assess the performance of GPT-4 in each category, Fisher’s exact test was implemented for each classification category (lower- vs. higher-order thinking, two-answer vs. single-answer, diagnostic radiology vs. nuclear medicine, nuclear medicine vs. radiation oncology, and radiation oncology vs. diagnostic radiology). All tests were two-tailed, and *p*-values less than 0.05 were considered significant. All *p*-values were nominal, and corrections for multiple comparisons were not performed. All statistical analyses were performed using R version 3.5.1 (R Foundation for Statistical Computing, Vienna, Austria).

### Ethical considerations

This study did not involve human subjects or patient data. All data used in this study are publicly available on the Internet. Therefore, the study was excluded from the consideration of the Institutional Review Board of Tohoku University.

## Results

### Overall performance of ChatGPT, GPT-4, and Google Bard

In the experiment, 103 questions from the JRBE 2022 were used. ChatGPT, GPT-4, and Google Bard correctly answered 40.8% [95% confidence interval (CI): 31.2%–50.9%], 65.0% (95% CI: 55.0%–74.2%), and 38.8% (95% CI: 29.4%–48.9%) of the questions, respectively. While previous studies have reported that LLMs may occasionally respond to multiple-choice questions without selecting an alternative, in this study, we chose at least one or two options for each question [5]. GPT-4 significantly outperformed ChatGPT by 24.2% (*p* < 0.001). GPT-4 also outperformed Google Bard by 26.2% (*p* < 0.001). No significant difference was observed between ChatGPT and Google Bard (*p* = 0.86).

**Fig. 1:**
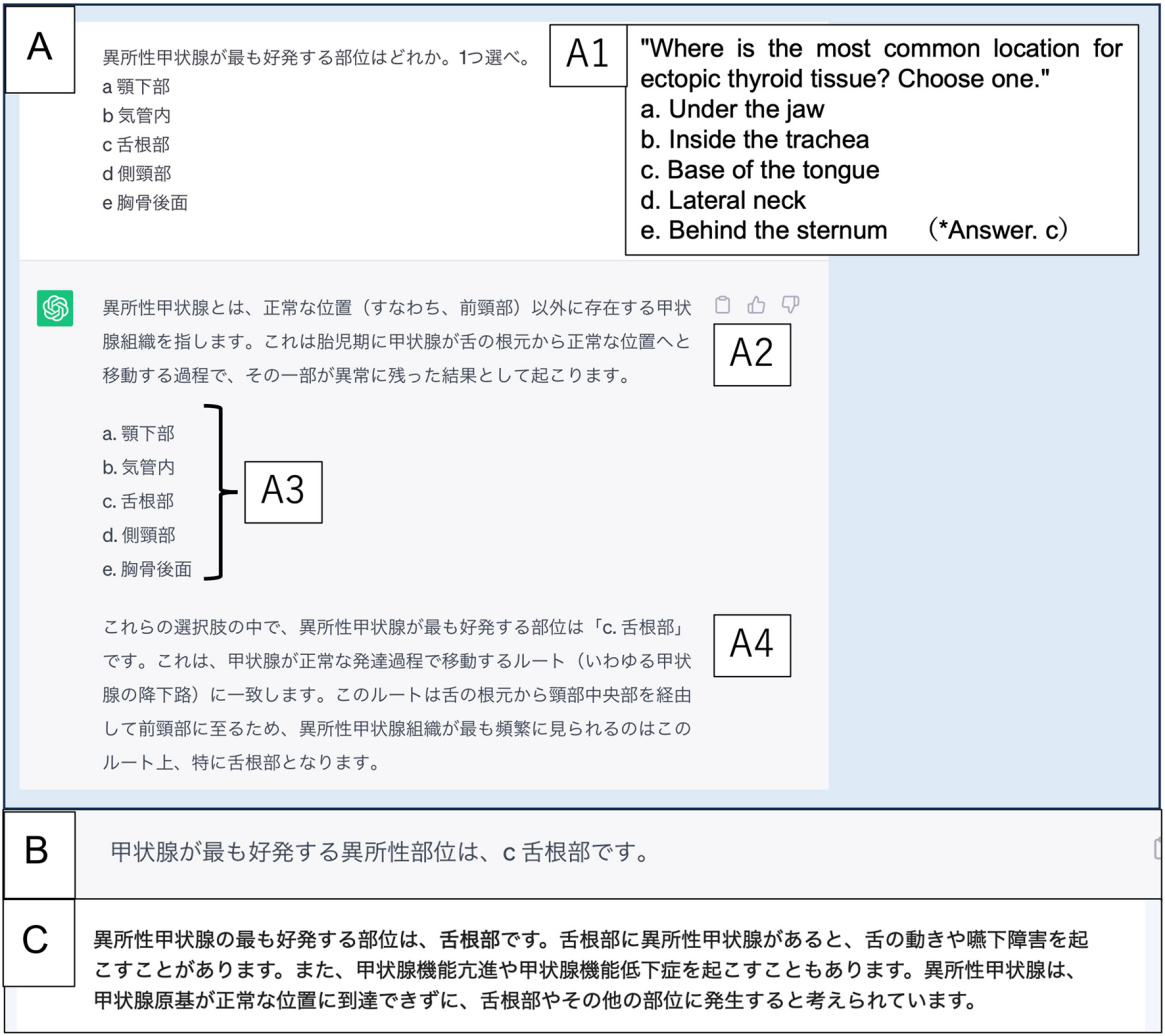
Response samples of correct answers generated by each LLM on a JRBE question. **A** Manually typed question into a prompt and response generated by GPT-4; A1: English version of (**A**). **B** ChatGPT response. **C** Google Bard response. The models’ responses varied in structure; most include an overview of the topic relevant to the question (A2), a representation of the alternatives (A3), and the answer and its justification (A4). Sometimes, the response simply states the answer (**B**); sometimes, the answer is generated with an explanation (**C**)

**Fig. 2:**
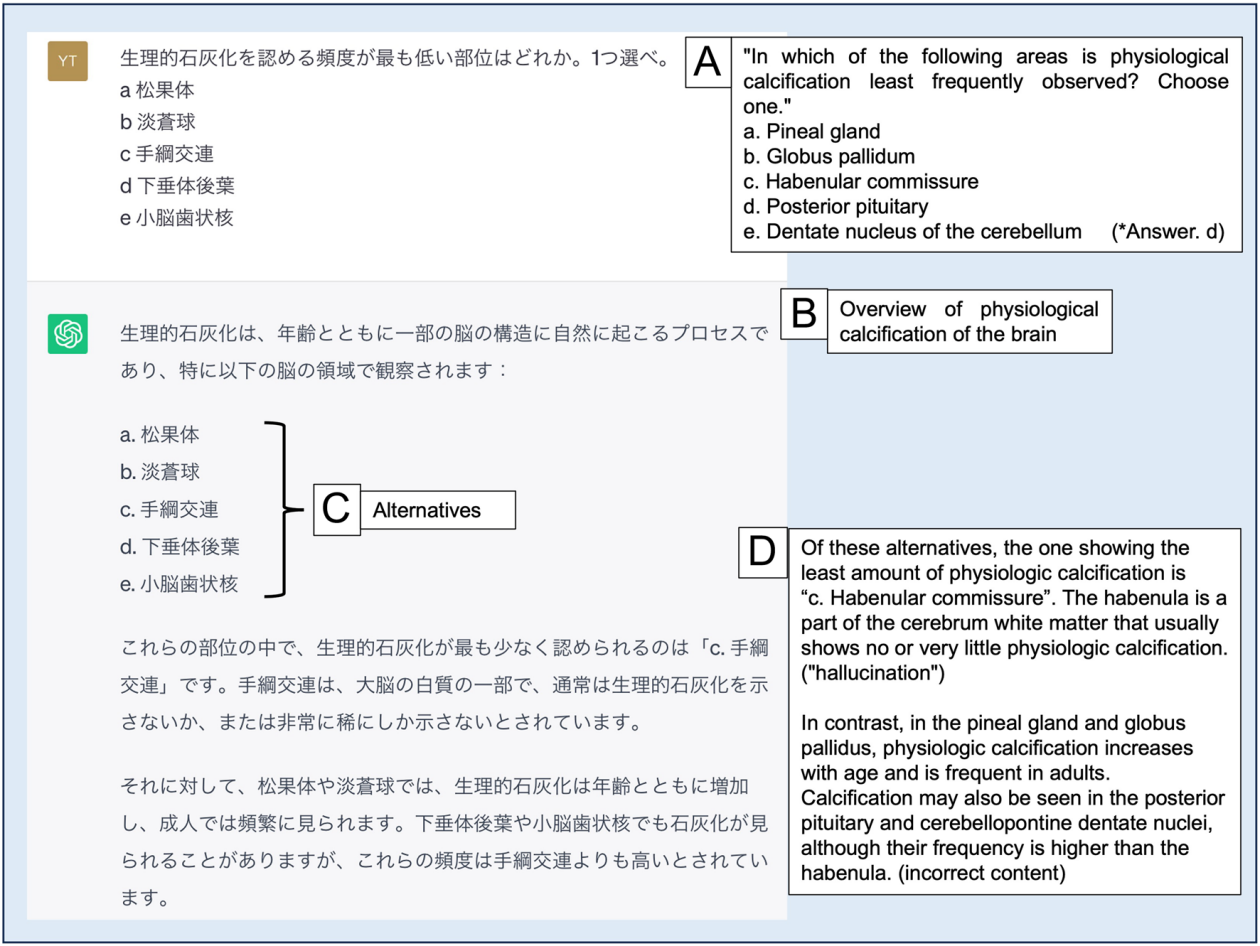
Response samples presenting “hallucination” generated by GPT-4 on a JRBE 2022 question. **A** Manually typed question into prompt and response; **A** English version of the question. The response is structured in the same way as that in Fig. 1. **B** Overview of the topic related to the question. **C** Representation of the provided alternatives; and **D** answer and its justification. In this response, a wrong answer and its justification are presented in a confident, convincing tone, which is called “hallucination”

Figure 1 depicts how GPT-4 correctly responded to a question regarding ectopic thyroid. Figure 2 depicts how GPT-4 replied incorrectly to a question concerning the anatomical structure of the brain, where physiological calcification is common. Although the explanation provided in the response is incorrect, the tone of the sentences is confident without a doubt, which represents an adverse phenomenon known as “hallucination” in LLMs (*p* = 0.86).

### Comparison of model performance on questions categorized by level of thinking

GPT-4 correctly answered 79.7% of the lower-order questions (*n* = 59), which was significantly higher than ChatGPT or Google Bard (*p* < 0.001). The proportion of correct responses to higher-order questions was not significantly different among the three LLMs.

### Comparison of model performance on questions categorized by question pattern

GPT-4 outperformed ChatGPT on the two-answer questions (52.9% vs. 11.8%, *p* = 0.023). A comparison between GPT-4 and Google Bard revealed no evidence of a difference between the two responses. When compared to the single-answer questions (*n* = 86), GPT-4 presented significantly better performance than ChatGPT (67.4% vs. 46.5%, p = 0.004) and Google Bard (67.4% vs. 39.5%, *p* < 0.001).

### Comparison of model performance on questions categorized by topic

GPT-4 correctly answered 93.3% of the questions in nuclear medicine (*n* = 15), outperforming ChatGPT (40%, *p* = 0.013) and Google Bard (26.7%, *p* = 0.004). GPT-4 also outperformed ChatGPT in terms of questions on radiological general knowledge (90% vs. 30%, *p* = 0.041). No significant differences were noted between the models for questions in diagnostic radiology and radiation oncology. Performance in interventional radiology could not be compared because of the limited number of questions (Table 1).

**Table 1:** Performance of ChatGPT, GPT-4, and Google Bard on Japan Radiology Board Examination, stratified by level of thinking, question pattern, and topic

Question type	No. of questions	Performance			*p* value		
		GPT-4	ChatGPT	Bard	GPT-4 vs. ChatGPT	GPT-4 vs. Bard	ChatGPT vs. Bard
All questions	103	67 (65.0, 55.0–74.2)	42 (40.8, 31.2–50.9)	40 (38.8, 29.4–48.9)	< 0.001	< 0.001	0.864
Level of thinking							
Higher-order	44	20 (45.5, 30.4–61.2)	16 (36.4, 22.4–52.2)	15 (34.1, 20.5–49.9)	0.502	0.267	> 0.99
Lower-order	59	47 (79.7, 67.2–89.0)	26 (44.1, 31.2–57.6)	25 (42.3, 29.6–55.9)	< 0.001	< 0.001	> 0.99
Question pattern							
Single-answer	86	58 (67.4, 56.5–77.2)	40 (46.5, 35.7–57.6)	34 (39.5, 29.2–50.7)	0.004	< 0.001	0.361
Two-answer	17	9 (52.9, 44.0–89.7)	2 (11.8, 1.5–36.4)	6 (35.3, 14.2–61.7)	0.023	0.450	0.134
Topic							
Diagnostic radiology	52	29 (55,8, 41.3–69.5)	22 (42.3, 28.7–56.8)	22 (42.3, 28.7–56.8)	0.169	0.121	> 0.99
Interventional radiology	3	1 (33.3, 0.8–90.6)	0 (0, 0–70.8)	0 (0, 0–70.8)	N/A	N/A	N/A
Nuclear medicine	15	14 (93.3, 68.1–99.8)	6 (40, 16.3–67.7)	4 (26.7, 7.8–55.1)	0.013	0.004	0.724
Radiation oncology	23	14 (60.9, 38.5–80.3)	11 (47.8, 26.8–69.4)	8 (34.8, 16.4–57.3)	0.505	0.077	0.505
General knowledge	10	9 (90, 55.5–99.7)	3 (30, 6.7–65.2)	6 (60, 26.2–87.8)	0.041	0.371	0.248

Data were provided as a number of correct answers. The percentages and 95% confidence intervals are shown in parentheses

### GPT-4 performance comparison between categories

GPT-4 performed better on lower-order questions compared to higher-order questions (79.7% vs 45.5%, *p* < 0.001). GPT-4 performed slightly better on single-answer questions than on two-answer questions (67.4% vs. 52.9%, *p* = 0.275). A comparison of the performance of GPT-4 between topics with an adequate number of questions (i.e., diagnostic radiology, nuclear medicine, and radiation oncology) revealed that GPT-4 performed significantly better in nuclear medicine than in diagnostic radiology (*p* < 0.001), but no difference was observed between nuclear medicine and radiation oncology (*p* = 0.0562) or diagnostic radiology and radiation oncology (*p* = 0.802).

## Discussion

Our study is the first to investigate the performance of ChatGPT, GPT-4, and Google Bard in the context of extensive expert-level knowledge and their application in Japanese clinical settings in radiology. The thoughtfully balanced question set honed by the JRBE committee enabled us to conduct overall and categorical assessments of the capabilities of the LLMs. GPT-4 outperformed ChatGPT and Google Bard in terms of overall performance, which is consistent with the findings of earlier research [4, 8, 9, 13, 15, 20, 22]. This finding, however, contradicts our initial hypothesis that Google Bard, using up-to-date data, would outperform GPTs, which do not utilize data beyond 2021, in addressing questions originating from the fast-growing field of medicine. This suggests that the use of the most recent two years of training data by Google Bard may not provide a significant advantage over GPTs. Because the questions in board examinations are supposedly based on medical knowledge that is well established from research and clinical practice, the latest data available for Google Bard may not necessarily aid in solving the questions. The overall performance of GPT-4 was 65%, slightly above the passing line, which was set at 65% as a point of reference. Although this accuracy seems lower than the reported accuracy of 80.7% for four-choice questions mimicking the diagnostic radiology board examinations in the United States and in Canada [8, 9], the JRBE questions may be more difficult because of the five-choice format with occasional two correct answers and its broader coverage of clinical areas, including diagnostic radiology, nuclear medicine, and radiation oncology.

GPT-4 outperformed the other models in terms of category-specific performance, particularly for lower-order or single-answer questions or questions in nuclear medicine (Table 2). Notably, in research using radiology board-style examinations in Canada and the United States, the superiority of GPT-4 over ChatGPT on lower-order questions has not yet been reported [9]. This could be attributed to the relatively small amount of medical training data in Japanese compared to that in English, as the majority of medical research and guideline databases, such as PubMed, Web of Science, and Scopus, are predominantly in English. In terms of topic differences, GPT-4’s superior performance in nuclear medicine may be attributed to the highest lower-question proportion of 100% in nuclear medicine, while the corresponding metrics were 84.25%, 69.69%, and 71.4% in diagnostic radiology, radiation oncology, and general radiology, respectively.

**Table 2:** GPT-4 performance comparison across questions categories

Question category	GPT-4’s performance	*p* value
Higher-order vs lower-order	45.5% vs 79.7%	< 0.001
Single vs two-answer question	67.4% vs 52.9%	0.275
Diagnostic radiology vs nuclear medicine	55.8% vs 93.3%	< 0.001
Nuclear medicine vs radiation oncology	93.3% vs 60.9%	0.0562
Diagnostic radiology vs radiation oncology	55.8% vs 60.9%	0.802

Statistical significance was calculated using Fisher’s exact test

Consistent with earlier research, the three models presented answers in confident language for both correct (Fig. 1) and incorrect responses (Fig. 2). Surprisingly, they behaved confidently although their responses differed from their previous choices for the same question (examples not shown). Such unfavorable responses entirely grounded in incorrect evidence or factual inaccuracies are commonly labeled as “hallucinations;” however, some advocate using “confabulations” as a neutral designation without implying malicious intent [5, 14]. Regardless of nomenclature, our findings support the idea that when using LLMs in clinical contexts, users must evaluate whether the model’s response is factually accurate and relevant to the topic, irrespective of the level of confidence.

When discussing approaches to improve the accuracy of LLM responses, ways to frame a prompt, often known as “prompt engineering,” should also be considered. Previous research on ChatGPT performance on the United States Medical License Examination, for instance, standardized the structure of questions entered into ChatGPT’s prompts [13]. According to Wang et al., on questions appearing in pharmacy licensing tests in Taiwan, ChatGPT answered more accurately for questions in English than in Chinese [24]. This may suggest that prompt engineering may include selecting a language to “communicate” with LLMs. Investigating the effects and impacts of different types of prompt engineering on the accuracy of LLMs is beyond the scope of this study and is currently under investigation.

This study had several limitations. First, the number of questions used was relatively small, which may have resulted in inadequate analysis, particularly for category-specific performance. Second, we did not use JRBE 2020/2021 but only JRBE 2022 because the training data used for ChatGPT/GPT-4 utilize data until September 2021, which may include JRBE 2020/2021. As LLMs update their training datasets, their performance on JRBE 2022 may also change quickly in the future. Third, no prompt engineering was executed in our study, which may have hindered the superior performance of LLM. Fourth, Google Bard, used in this study, is based on PaLM2, which is not fine-tuned for medical purposes, unlike Med-PaLM 2. Thus, we may have underestimated the potential of future LLMs trained using medical data. Fifth, the answers used for LLM performance evaluations were not official. The possibility of answer inaccuracies was minimized through consensus among the three radiologists. Finally, we used a provisional passing threshold for the JRBE because it was not officially declared. To evaluate the performance of LLMs, we need to compare their accuracies with those of radiology residents who had just passed the JRBE.

In conclusion, GPT-4 scored 65% when answering Japanese questions from JRBE, outperforming ChatGPT and Google Bard. This highlights the potential of using LLMs to address advanced clinical questions in radiology in Japan. Nevertheless, checking the generated responses is crucial to prevent potential harm to patients and radiologists.
